# More than a free snack: benefits of mobilized resources and socialization for pediatric healthcare workers

**DOI:** 10.3389/frhs.2026.1958370

**Published:** 2026-09-14

**Authors:** Thomas B. Ayres, Kristin L. Ray, Aimee M. Hoflander, Angela L. Myers

**Affiliations:** 1 Center for Wellbeing, Children’s Mercy Kansas City, Kansas City, MO, United States; 2 Department of Pediatrics, University of Missouri-Kansas City School of Medicine, Kansas City, MO, United States

**Keywords:** energy, engagement, health care workers, intervention, perceived organizational support, well-being, work breaks and micro-breaks

## Abstract

**Introduction:**

Addressing the burnout epidemic in the healthcare workforce is a primary concern of the modern healthcare organization and recognized across researchers and leaders of nations. This study aims to test whether a mobilized micro-break intervention, the Wellbeing on Wheels (WOW) Cart, improved health care worker (HCW) energy perceived organizational support and work engagement.

**Methodology:**

This study was conducted at an independent children's hospital in the midwestern United States. Between August and September 2024. A total of 367 pediatric HCWs completed our survey. IBM's SPSS was used to analyze the data using *t*-tests, ANOVAs, linear regression, and polynomial regression to test our hypotheses and *post-hoc* analyses.

**Results:**

The results of this study show that energy (*t* = 32.35, *p* < .01, Cohen's *D* = 1.33), perceived organizational support (*t* = 17.40, *p* < .01, Cohen's *D* = .92), and work engagement (*t* = 20.56, *p* < .01, Cohen's *D* = 1.08) significantly increased following a visit by the WOW Cart. Further, we found that both changes in energy (*β* = .44, *p* < .01) and perceived organizational support (*β* = .32, *p* < .01) predicted increases in work engagement (*R*^2^ = .35, *p* < .01). Our *post-hoc* analyses revealed nurses had a greater energy benefit [(*F*_(2, 276)_ = 2.96, *p* < .05] compared to nurse practitioners (*M*_diff_ = .82, *p* < .05) and physicians (*M*_diff_ = .55, *p* < .05), but no difference between job roles for perceived organizational support [(*F*_(3, 281)_ = .47, *p* = .71] and work engagement [(*F*_(2, 283)_ = 1.77, *p* = .15]. Further, we found an exponential increasing benefit from the degree of change in energy for work engagement (coeff. = .53, *t* = 2.949, *p* < .05). Additionally, we found a linearly increasing benefit from the degree of changes in perceived organizational support for work engagement (coeff. = −1.88, *t* = −4.433, *p* < .01).

**Conclusions:**

This study highlights that resource-based micro-breaks may be a viable option for effective HCW well-being intervention. Moreover, interventions can be brought to point-of-care areas. Additional research should determine how long these improvements last.

## 1 Introduction

Effective well-being interventions for health care workers (HCWs) are critical in the current work climate (1–3). The healthcare workforce has been plagued with well-being barriers since long before the COVID-19 pandemic (3). Indeed, former surgeon general Vivek Murthy stated, “The Nation's health depends on well-being in our workforce. Confronting the long-standing drivers of burnout among our healthcare workers must be a national priority” (4). Further, researchers have advocated for a focus on creating and sustaining a culture that supports well-being and inclusion, building personalized resilience, and improving HCW well-being through workplace improvement. These practices empower HCWs to realign their purpose with their mission of caring for others (2, 3). The continued evidence showing the poor state of well-being for HCWs emphasizes the need for effective interventions to combat burnout and build a culture of well-being (1–6).

We tested the effectiveness of a resource providing a micro-break intervention for HCWs in their point-of-care areas. We contribute to research on well-being interventions by grounding our intervention in a foundational stress theory, conservation of resources (COR) theory (1, 7). Through our application of COR, we provide a rationale for why a micro-break intervention providing external resources in the form of small nutritional items combined with positive socialization can improve internal resources of energy and perceived organizational support (POS) and, ultimately, work engagement.

Micro-breaks are short, usually unscheduled, periods of respite separate from institutionally scheduled breaks. Micro-breaks last up to 10 min and can occur between or during work tasks (8). Researchers have found that micro-breaks can be beneficial for work engagement (9, 10), decreasing fatigue and low energy (9–12), improving neuromusculoskeletal health (13), increasing work stress recovery (14, 15), reducing unethical behavior (16), and improving well-being (8, 17). In HCWs, micro-break interventions that included reminders to take short stretch breaks have been shown to support musculoskeletal health in surgeons (13), but not musculoskeletal endurance or decreased procedure time (18). In non-HCWs, social and nutritional micro-breaks were positively related to engagement, with social micro-breaks also being beneficial for reducing end-of-day fatigue (9, 10). Our micro-break intervention builds on research studying the benefits of nutritional and social micro-breaks. As our micro-break was organizationally initiated, we also studied the impact of the combined social and nutritional micro-break on POS.

Multiple studies of micro-breaks use COR theory as a framework to describe the benefits of micro-breaks (9–11). This is appropriate given that many micro-break interventions are concerned with facilitating or providing access to resources that the employee lacks or has depleted. Other theories and frameworks of well-being do not specifically attend to the dynamic changes in resources that employees experience. Job-demands resources theory focuses on the interplay between demands experienced and resources available and is more appropriate for examining well-being while work is going on and not as much during respite (19). The effort-recovery model provides a good rationale for why micro-breaks are needed, i.e., to provide brief interruption to the ongoing activation of stressors, but lacks a structure to describe why specific micro-breaks are beneficial beyond just interrupting work stressors (20). Recovery as self-regulation is likely the next best framework compared to COR in arguing that micro-breaks could be necessary for up-regulating energy to get through the next work task or for down-regulating stress activation after a difficult work event (21). However, recovery as regulation for this study describes self-motivated micro-breaks (i.e., up- or down-regulate) well but breaks down when considering third party or organizationally initiated micro-breaks where a person may not feel driven for up- or down-regulation. Therefore, we chose COR to describe how the micro-break intervention we provided facilitates access to nutritional and social resources that in turn can facilitate the resources investment process of COR and improve both resources and well-being of HCWs.

COR theory principle 2 and its corollaries 1–3 can explain how a micro-break intervention within the workplace can support HCW energy, POS, and work engagement (7, 22). COR theory posits that employees have a finite number of resources available to them and that the degree this pool of resources is depleted or threatened to be depleted determines an employee's level of well-being (7). Employees experience resource threat when resources are depleted or when they encounter situations where they must use available resources to meet demands of work. As depletion occurs, resources need to be replenished. Principle 2 of COR theory, known as the principle of resource investment, is related to replenishing resources. It explains that employees must invest resources to gain resources and protect themselves from losing resources, ultimately enabling a resource-rich environment and state of being to support well-being and performance (7, 22). Corollaries 1–3 of COR address additional key aspects of the dynamic process of resource investment: (1) individuals with more resources are better positioned for resource gains, (2) initial resource gains lead to future resource gains, and (3) lack of resources leads to defensive attempts to conserve remaining resources (22). Therefore, to support HCW well-being, organizations should find ways to provide necessary resources that can be used in the process of resource investment to build additional resources or protect against resource loss. Further, organizations need to identify avenues for counteracting the defensive attempts to conserve remaining resources when resources are low.

In line with Principle 2 of COR theory and corollaries 1–3, it was paramount that we bring the intervention directly to the HCWs' point-of-care areas to minimize required resource investment to engage with the intervention. Maximizing the balance of resources available to the employees by bringing them to their places of work should position them better for resource gains (Corollary 1), and this initial increase in resources provided by the micro-break intervention should promote future resource gains (Corollary 2). Finally, decreasing barriers to using the resources such as bringing them directly to the point-of-care area should reduce potential defensive attempts to conserve remaining resources (Corollary 3) (22). Thus, the micro-break intervention needed to be brief and appropriate for HCWs' point-of-care areas. As a result of these criteria, we structured our micro-break intervention around a mobilized cart containing nutritional resources and accompanied by organizational representatives focusing on providing positive socialization. This micro-break intervention allowed us to go directly to HCWs' area of work and target improving HCW energy, POS, and work engagement.

There are many different types of resources. Researchers have broadly defined resources as anything of value to the employee that helps them accomplish a goal (7, 22). Resources may also be something that helps build other resources. Resources can be labeled based on where they reside: within the employee (i.e., personal resources, attitudes) or in the environment (i.e., tangible items, social interactions). Personal resources also have been labeled as related to the person (i.e., energy) or the workplace (i.e., POS) (22). Therefore, in line with Corollary 2 of COR theory, we combined providing two external resources, small nutritional items and socialization, with targeted increases in two personal resources, energy and POS (22). Thus, the small nutritional items and socialization were the basis of our intervention to provide environmental resources to promote accumulation of personal resources.

Energy is a personal resource that aids employees in resource investment and is one of the most critical resources that, when depleted, leads to defensive attempts such as decreased work engagement to conserve remaining energy (23). Researchers studying how to build employee energy at work emphasize the need for providing a framework that supports autonomy and social support during breaks and micro-breaks (24). Therefore, we made an assortment of small nutritional items a part of our intervention to allow HCWs autonomy in choosing items to enjoy free of charge during their break or micro-break. Further, socialization can lead to relational energy that is available for the HCW to draw upon (25). Providing these external resources in the form of small nutritional items, autonomy of choice, and socialization should facilitate gains in a personal resource of energy (COR Corollaries 1 & 2) (22). Therefore, we propose:

Hypothesis 1: Energy will increase from pre-WOW Cart arrival to post-WOW Cart.

Personal resources also include employee attitudes towards the workplace that support their well-being and engagement. Thus, our intervention also targeted the well-studied resource of POS. Organizational support theory posits that based on actions of the organization, employees develop a perception of the degree the organization cares about their work and their well-being (26). These perceptions then determine the degree the employee is committed to and engages within the organization. Indeed, increases in POS are associated with increases in well-being (26, 27). Building POS is characterized by changing perceptions of the degree the organization values and supports them.

One way that HCWs may receive information concerning the degree the organization values and supports them is from the other people around them. Socialization, the process of interacting with others, has been well studied within work breaks and micro-breaks (9). In general break settings, socialization with high autonomy during lunch has been shown to reduce end-of-work fatigue (10) and increase vitality (12). Within micro-breaks, socialization breaks have been shown to be positively related to work engagement (10, 11), sales performance, and positive affect (9), suggesting that socialization at work can lead to relational energy that can be used in work (25). Intriguingly, socialization in micro-breaks has also been shown to be positively related to end-of-day fatigue (11). This may be explained by results showing that employees who start their day higher in fatigue engage in fewer social micro-breaks (11). We argue that short socialization breaks focused on support and communicating the value of HCWs' work by representatives of the organization can facilitate improvements in perceptions of value to the organization. In other words, providing an external resource (i.e., socialization) facilitates gains in a personal resource (i.e., POS) in line with Corollaries 1 & 2 (22). We propose:

Hypothesis 2: HCWs' perceptions of organizational support will increase from pre-WOW Cart arrival to post-WOW Cart.

Employees who experience gains in resources are better positioned to address stressors and maintain their well-being (7, 22). Further, greater resource levels should reduce the drive to engage in defensive attempts to conserve remaining resources (Corollary 3) (22). Thus, HCWs with more resources are less likely to reduce work effort to conserve and protect their well-being. Therefore, HCWs should be more willing to invest into their work and, as such, engagement should increase. Engagement is a positive connection to work characterized by vigor, dedication, and absorption (28). We posit that the gains in resources facilitate better investment in work that leads to increases in engagement according to the principle of resource investment and Corollary 2 (7, 22). We propose:

Hypothesis 3: Engagement will increase from pre-WOW Cart arrival to post-WOW Cart.

Hypothesis 4: Changes in (a) energy and (b) POS will positively predict changes in engagement.

## 2 Method

### 2.1 The cart

The WOW Cart is a four-tier shelving cart that contains nutritional items including drinks (e.g., flavored water, soda, energy drinks), snack items (e.g., chips, beef sticks, fruit bars), and personal care items (e.g., comfy socks, lip balm, fidget devices). Center for Wellbeing team members and volunteers managed and accompanied the cart to different inpatient and ambulatory areas of the hospital on a bi-weekly cadence and to targeted areas of the hospital after acute critical events (e.g., trauma activation or patient death). The central message they used to start each encounter was that their purpose in being there was to communicate that the work HCWs are doing every day, hour, or minute in their respective roles and point-of-care areas is seen, appreciated, and valued. Then they engaged in conversations discussing how the HCWs' days/weeks were going, sharing messages of leaderships awareness and many times requests initiating a WOW Cart visit, and informing them about other resources available from the organization's Center for Wellbeing.

### 2.2 Procedure

To test our intervention, we recruited participants through handouts and signs placed on the WOW Cart. The survey provided a study information letter, ascertained consent, and asked participants to recall their energy, POS, and engagement before the WOW Cart arrived to their work area. Then the survey asked them to report their current energy, POS, and engagement after the WOW Cart visited. Finally, participants reported demographic characteristics and could choose to enter their email for a raffle to win a branded t-shirt at 1:10 odds. This study was determined exempt from full review by our institution's ethics committee.

### 2.3 Participants

Data collection spanned from August 6th to September 30th, 2024. In that period, the WOW Cart was implemented 29 times. A total of 431 attempted the survey, and 367 individuals completed our survey (complete rate = 92%). Thirty HCWs were repeat responders. We removed their second responses to keep the variance in our dataset consistent with HCWs first time reporting their experience with the WOW Cart. Participants were on average 33.09 years old (SD = 10.71). They were 89.1% (*N* = 327) female, 10.6% (*N* = 39) male, and 0.3% (*N* = 1) non-binary. Race/ethnicity included 81.2% (*N* = 298) Caucasian or white, 7.6% (*N* = 28) African-American, 1.9% (*N* = 7) Asian-American or Pacific Islander, 6.3% (*N* = 23) Latino, 0.3% (*N* = 1) Native American or American Indian, 0.8% (*N* = 2) other, and 1.9% (*N* = 7) preferred not to report race. Key clinical job roles represented included nurses (58.3%, *N* = 214), physicians (12.5%, *N* = 46), medical assistants and nurse technicians (8.7%, *N* = 32), and nurse practitioners (3%, *N* = 11). The remaining 17.5% (*N* = 64) of participants were administrative, research, and environmental services personnel. Most participants (80.7%, *N* = 296) were full-time equivalent employees (.75 FTE or higher) and 19.3% (*N* = 71) part-time (less than.75 FTE).

### 2.4 Measures

Our study used single-item measures for all three primary constructs. Historically, single- item measures have been criticized for the inability to assess internal consistency reliability and the breadth of measurement that is lost compared to longer measures of the same construct (29). However, despite these criticisms, published research on single-item measures shows comparable reliability and validity as longer measures (30). One challenge in implementing single-item measures in field studies of organizational research is the inability to include the longer measures of the same constructs needed for most methods of assessing reliability (31). The primary method for assessing reliability without having a longer measure is test-retest reliability. This method was not an option for our study because our measurements of the constructs referenced different time periods, so it would not be possible to ascertain whether low correlations were due to unreliability or the differences in changes associated with intervention. Therefore, we relied on the reliability evidence presented in the development of previously published measures and the content validity of our POS item, two practices supported by literature on single-item measurement (29–31).

#### 2.4.1 Energy

We used the single-item pictorial scale of human energy (32). This scale asks participants to indicate which of seven images best describes their energy level, from depleted to full of energy, as depicted by levels of the charge of a battery. This measure has previously been shown to be consistently sensitive to changes in energy, similar to longer published measures of energy (32).

#### 2.4.2 Perceived organizational support

We used the item “I feel my work is valued” to measure POS via feelings of work being valued. Participants responded using a 1 (*Strongly disagree*) to 5 (*Strongly agree*) Likert-type scale. The content validity of the item was evaluated by a panel of eight subject matter experts and agreed upon to represent POS.

#### 2.4.3 Engagement

We used a single-item measure of engagement that asks participants to select their level of engagement on a scale from 0 (*lowest possible level of work engagement*) to 10 (*highest possible of level of work engagement*) (33). This measure has been shown to be reliable using correction for attenuation methods when using the strong correlations with the Utrecht Work Engagement Scales: the UWES 3, UWES 6, and UWES 9. The convergent validity evidence from strong correlations with all three forms of the UWES provides support for using a single-item to measure a normally multi-faceted construct (33).

## 3 Results

Means, standard deviations, and correlations for study variables are shown in Table 1. We ran paired samples *t*-tests to test hypotheses 1–3 and linear regression to test hypothesis 4. Hypothesis 1–3 stated that energy, POS, and engagement would be significantly higher post-WOW Cart intervention compared with pre-WOW Cart intervention. We found that post-wow cart ratings of energy (*t* = 32.14, *p* < .01, Cohen's *D* = 1.33), POS (*t* = 16.93, *p* < .01, Cohen's *D* = .85), and engagement (*t* = 20.24, *p* < .01, Cohen's *D* = 1.86) were significantly higher than pre-wow cart ratings. Therefore, hypotheses 1–3 were supported.

**Table 1:** Means, standard deviations, and correlations for study variables.

Variable	Mean	SD	1	2	3	4	5	6
Pre-WOW cart intervention								
1. Energy	3.99	1.30						
2. Perceived organizational support	3.71	0.80	.11*					
3. Engagement	6.54	2.03	.39**	.35**				
Post-WOW cart intervention								
4. Energy	6.26	0.79	.25**	.06	.15*			
5. Perceived organizational support	4.48	0.65	.01	.33**	.05	.27**		
6. Engagement	8.53	1.25	.10	.14**	.44**	.56**	.32**	

*N* = 367.

* *p* < .05.

** *p* < .01.

Hypothesis 4 posited that changes in (a) energy and (b) POS would positively predict changes in work engagement (Table 2). We found that changes in energy (*β* = .44, *p* < .01) and POS (*β* = .33, *p* < .01) positively predicted changes in work engagement (*R*^2^ = .36, *p* < .01). Therefore, hypotheses 4a and 4b were supported.

**Table 2:** Regression results for changes in energy and perceived organizational support predicting changes in work engagement.

Variable	*B*	*β*	*R* ^2^
Constant	.04		
Change in energy	.61	.44**	
Change in perceived organizational support	.73	.33**	
			.35**

*N* = 367.

** *p* < .01.

### 3.1 *Post-hoc* analyses

We ran *post-hoc* analyses to: (1) examine whether the WOW Cart benefited physicians, nurses, or nurse assistant/technicians similarly or had differing effects according to job roles, (2) test whether higher initial resource levels in energy and POS contributed to larger resources gains, and (3) test whether similar degrees of resource change correspond to the same benefit for work engagement. Our one-way ANOVA revealed significant differences between job roles for changes in energy [(*F*_(3, 273)_ = 3.18, *p* < .05], but not for POS [(*F*_(3, 272)_ = .25, *p* = .78] or work engagement [(*F*_(3, 280)_ = 1.67, *p* = .17]. Fisher's Least Significant Difference (LSD) *post-hoc* tests revealed that nurses had a larger change in energy from pre- to post-Wow Cart ratings compared with nurse practitioners (*M*_diff_ = .81, *p* < .05) and physicians (*M*_diff_ = .54, *p* < .05). Therefore, the WOW Cart may be most beneficial for nurses regarding energy but similarly beneficial for each job role for POS and work engagement.

To test whether higher initial levels of resources contributed to larger resource gains, we conducted *t*-tests comparing changes in energy and POS for our sample between those with starting levels below the mean and those above the mean for each resource. Those with initial energy or POS below the mean had significantly larger changes between pre- and post-Wow Cart ratings for energy (*t* = 15.30, *p* < .01, Cohen's *D* = 1.73) and POS (*t* = 14.20, *p* < .01, Cohen's *D* = 1.59) (22). These findings seem to contradict corollary 1 of COR, those with more initial resources are better positioned for gaining additional resources. However, that is only when viewing personal resources alone and not recognizing that our intervention provided resources for the employees. In this way, corollary 1 is supported in that everyone no matter their personal resource level is better positioned for resource gain because of the resources provided to them. Additionally, these findings could reflect the reduction of resource investment required to build resources through our intervention by bringing them directly to work areas. Put another way, reducing the amount of resources required for resource investment by providing external resources could be a mechanism for equalizing the initial availability of resources to then allow for resource gain (22). Thereby the intervention may have been able to benefit those who needed it most (i.e., those with lowest initial resources). Alternatively, there could be a ceiling effect where the intervention could increase energy and POS to only a certain degree and our structure helped all participants get closer to that ceiling. This effect could explain the smaller standard deviation for post-WOW Cart visits compared to pre-WOW Cart visits. Or in other words, the WOW Cart brought HCWs with more varying levels of starting energy and POS to more similar levels of post-energy and POS.

Because our regression analyses to test hypothesis 4 used change scores, similar degrees of change at any level of the scale for energy and POS were treated similarly. However, to investigate whether experiencing the same degree of resource improvement benefits work engagement depending on starting level, we chose to run polynomial regression-based response surface analysis (RSA) to compare benefits to work engagement from similar levels of changes in energy or POS at varying points of the measurement scale (i.e., low end vs. high end). RSA is typically used to test congruence hypotheses between two different constructs (*X* & *Y*) on an outcome of interest (*Z*) (34, 35). We ran two models testing the combinations of differences in energy and POS on work engagement. We entered pre-energy as our *X* and post-energy as our *Y*, with changes in work engagement as our *Z*. We repeated this structure for POS. Therefore, our line of congruence shows when HCWs pre- and post- ratings reflect the same level of energy or POS. We are interested in the line of incongruence and other combinations of incongruence that show when HCWs pre- and post- ratings of energy or POS differ. RSA tests for a linear and curvature slope on the line of congruence and line of incongruence.

Our RSA results showed that the curvature of the line of incongruence was significant for energy (coeff. = .56, *t* = 3.110, *p* < .01) and the linear line of incongruence was significant for POS (coeff. = −1.93, *t* = −4.433, *p* < .01). For energy, a significant curvature for the line of incongruence suggests that the changes from pre- to post- ratings for work engagement exponentially increases as the degree of incongruence increases (Figure 1). If we look at the plotted points for describing the relationship between pre- and post-energy with changes in engagement, we see that where the starting point of incongruence, or pre-WOW Cart rating of energy, takes place on the scale of measurement matters for the degree of change observed for work engagement ratings. Specifically, the same degree of incongruence relates to larger changes for work engagement when pre-WOW Cart energy rating is higher (Table 3). For POS, a significant linear line of incongruence suggests that as the degree of incongruence increases, there is a similar degree of change in ratings for work engagement (Figure 2). When we look at plotted points for POS, we see a very similar change in ratings for work engagement for the same degree of incongruence at varying starting points on pre-WOW cart ratings of POS (Table 4).

**Figure 1:**
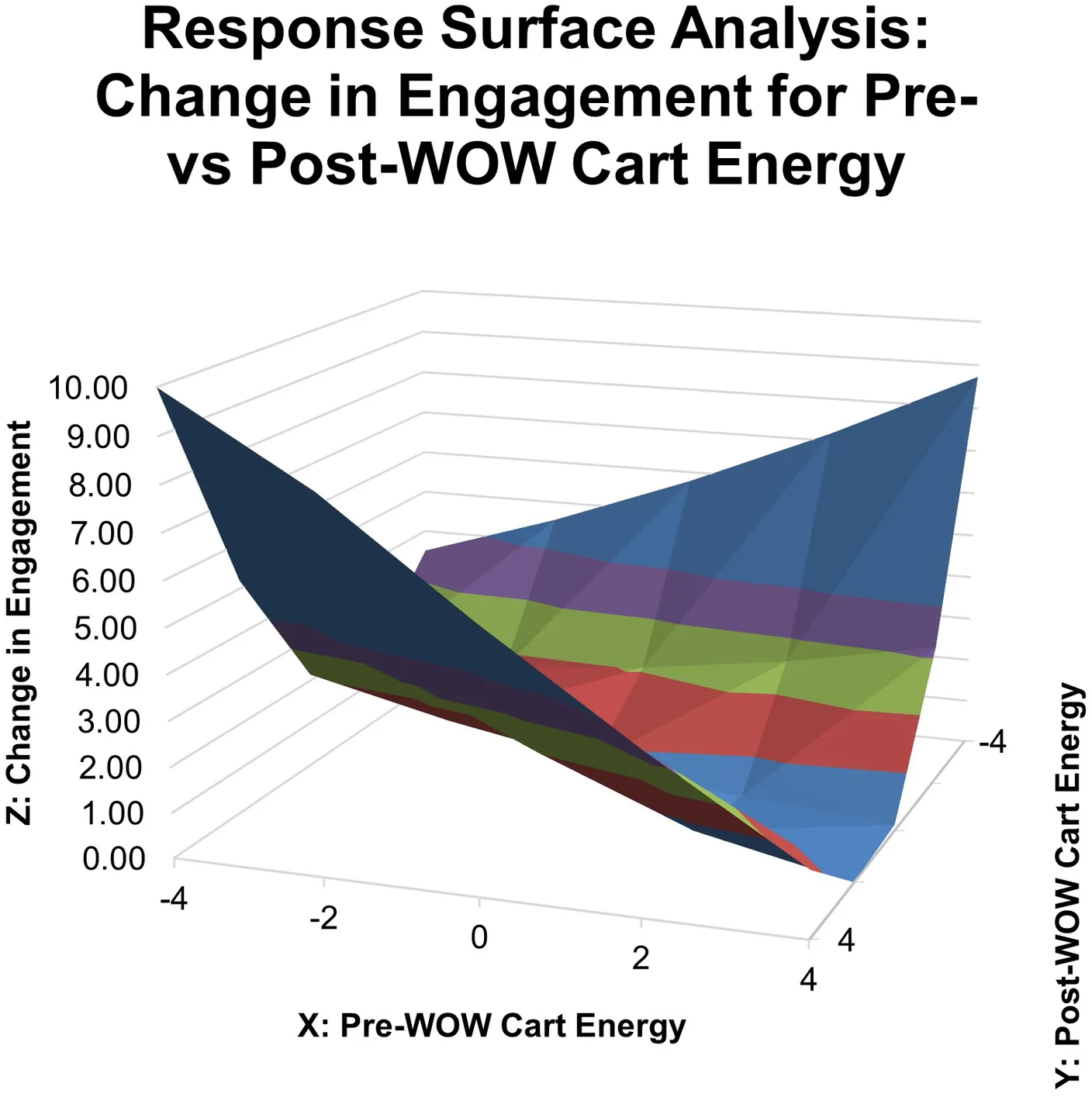
RSA predicting changes in engagement by incongruence of pre- post-WOW cart energy (35).

**Table 3:** Points to plot for response surface analysis for pre- post-WOW cart energy predicting changes in work engagement.

Predictors	Centered Scores	*X*: Pre-WOW cart energy				
		−4	−2	0	2	4
*Y*: Post-WOW cart energy	4	10.67	8.11	5.72	3.49	1.43
	2	5.17	3.52	2.03	0.70	−0.45
	0	2.15	1.39	0.81	0.39	0.14
	−2	1.59	1.74	2.06	2.54	3.20
	−4	3.50	4.56	5.78	7.17	8.73

*N* = 367. Diagonal is line of congruence (*X* = *Y*); below *X* > *Y*; above *X* < *Y*.

**Figure 2:**
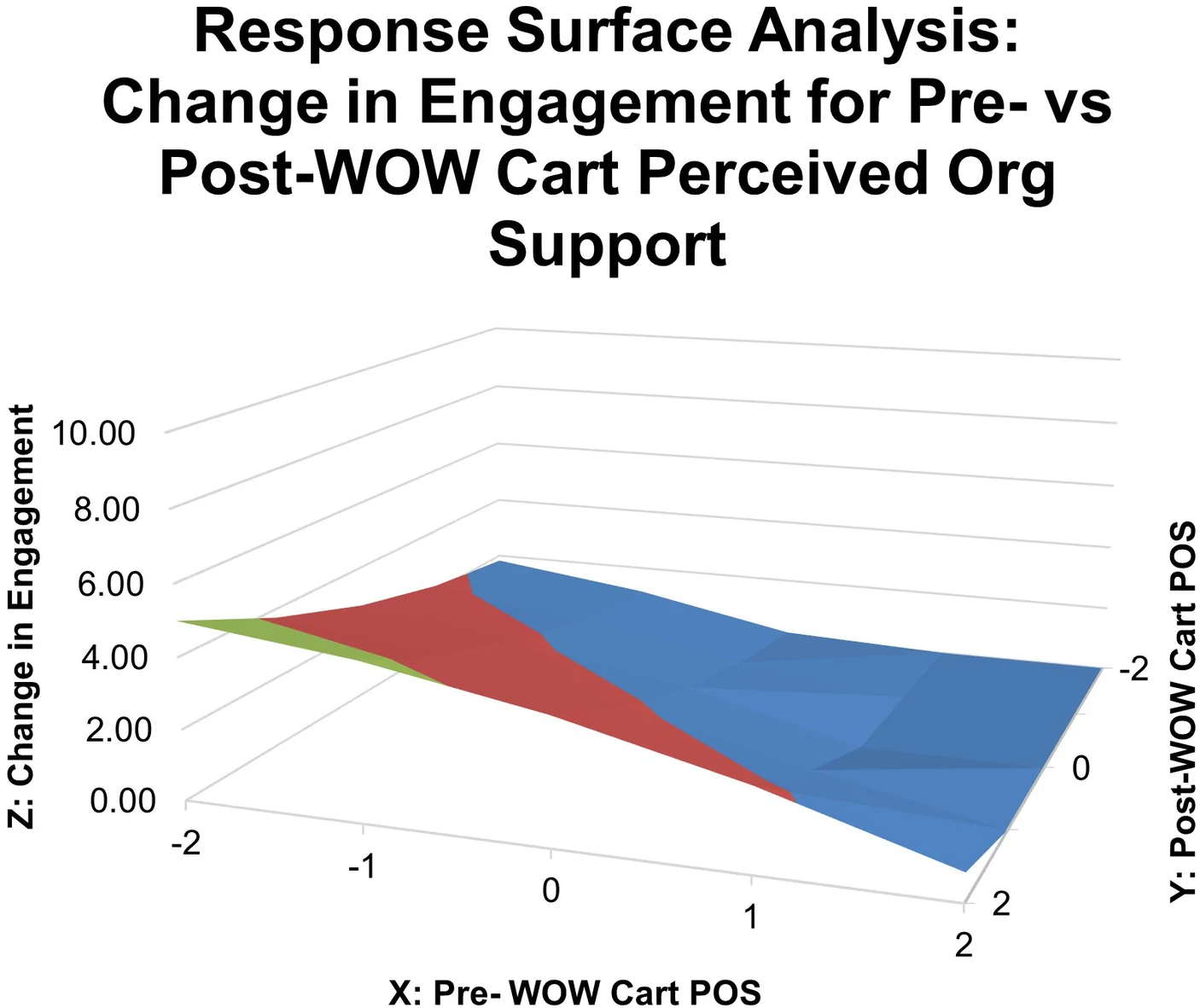
RSA predicting changes in engagement by incongruence of pre- post-WOW cart perceived organizational support.

**Table 4:** Points to plot for response surface analysis for pre- post-WOW cart POS predicting changes in work engagement.

Predictors	Centered Scores	*X*: Pre-WOW cart POS				
		−2	−1	0	1	2
*Y*: Post-WOW cart POS	2	4.99	4.43	3.54	2.32	0.78
	1	3.74	3.16	2.25	1.01	−0.55
	0	2.80	2.19	1.26	0.00	−1.58
	−1	2.16	1.53	0.58	−0.70	−2.31
	−2	1.82	1.17	0.19	−1.11	−2.74

*N* = 367. Diagonal is line of congruence (*X* = *Y*); below *X* > *Y*; above *X* < *Y*.

## 4 Discussion

We tested a micro-break intervention as a mechanism to support two personal resources and work engagement. Drawing from COR theory, we hypothesized that providing external resources through small nutritional items and socialization through the WOW Cart would facilitate resource investment for HCWs to build personal resources of energy and POS, respectively (7). Further, we posited that these increases in resources would contribute to an increase in work engagement. We found that on average HCWs' energy increased 28%, POS increased 15.6%, and work engagement increased 16.8% after a visit by the WOW Cart. Therefore, we found support for the resource investment process of COR and its corollaries in HCWs (7, 22). These findings provide several theoretical and practical implications.

### 4.1 Theoretical implications

Our study provides some support for the COR principle of resource investment and related corollaries 1–3 (7, 22). Through the structure of the WOW Cart, we posit that bringing the intervention to the point-of-care may have reduced the costs of resource investment to maximize the gains (Corollaries 1–2) and decreased defensive attempts to protect remaining resources and disengage (Corollary 3) (7, 22). Further, our *post-hoc* analyses for personal resources showed that those starting lower in energy and POS reported greater gains, suggesting our intervention could help reverse the resource loss process. In sum, these findings provide initial support that the availability of externally provided resources and removal of a barrier to investment by bringing the resources to point-of-care areas can contribute to improvements of ratings in the personal resources we studied, namely energy and POS (Corollaries 1–2). However, there may still be a benefit to encountering the WOW Cart with higher initial personal resources. Our *post-hoc* analyses provided insight that the WOW-Cart intervention may benefit those who need it most evidenced by larger changes in energy and POS for those who had pre-WOW Cart ratings below the median. However, regarding the benefit of changes in energy and POS for work engagement, our response surface analysis (RSA) observed that when comparing HCWs with similar degrees of change in personal resources of energy and POS, those who started with higher ratings of initial resources had a larger change in their work engagement scores. These findings support COR Corollary 1 in that the HCWs with a higher starting level of resources may be better positioned to make use of their resource gains to benefit other areas such as work engagement.

Second, we add to the literature on micro-breaks and provide support for how a micro-break intervention can be effective for HCWs. Previous literature on micro-breaks examined descriptive reports of behavior without intervention (8). Our reported findings of increased POS and energy showed that micro-breaks may be a crucial mechanism for creating and supporting a culture of well-being for HCWs (1, 3, 36).

### 4.2 Practical implications

We also can provide some practical advice based on this study. First, improving HCW energy and building a culture of well-being at an organization does not require extreme measures and can be a shared endeavor. We provide support for a small, brief intervention implemented by the organization that may significantly improve HCWs' energy and their perceptions of the organization supporting them. This intervention answers calls for organizations to take a shared responsibility approach to well-being (1, 5).

Second, we emphasize the importance of bringing the intervention to the HCWs' point-of-care areas. In line with COR theory, we identified that bringing external resources to the point-of-care areas would be necessary to support the initial availability of resources without requiring expenditure of their current resources as well as to kickstart the resource gain process to facilitate future resource gains, operationalizing Corollaries 1 and 3 (7, 22). We posit that this is key for starting the resource change process in a positive manner by reducing potential resource investment requirements associated with interventions. Our *post-hoc* analyses provide initial support that providing these external resources as a baseline might be especially important for HCWs starting with fewer resources because they reported greater gains. This supports that providing these external resources was enough to counteract Corollary 3, which posits that lack of resources leads to defensive attempts to conserve remaining resources.

Third, the combination of nutritional items with positive socialization was key. The WOW Cart visits were not solely a tangible external resource intervention but also a socialization intervention. Both nutritional items and socialization have been connected to improving employee energy (23–25). We found evidence that when implementing interventions to support HCWs, providing a multi-faceted approach does not need to be complicated. Pairing personalized social connection with the nutritional items enables a multi-faceted approach to impact HCW well-being and we found support that it impacts multiple personal resources.

### 4.3 Limitations and future research directions

Our study is not without limitations. We share five that must be considered when interpreting our results. First, our methodology was designed to be as minimally invasive on the workday of HCWs as possible. As such, we used single-item measures and collected data at a single time point asking participants to recall their energy and perceptions of organizational support prior to the WOW Cart arrival. This retrospective study design introduces recall bias and common method bias that causes some concern for potential inflation of the intervention effects. We chose to conduct measurements immediately after a visit form the WOW Cart to shorten the time to recall and mitigate memory decay and reconstruction. Future research would benefit from multi-modal data collection like biometric devices to remove the influence of common method bias in evaluating the impact of well-being interventions.

Second, as stated above, single-item measures have historically been considered inferior because of potential deficits in construct breadth and inability to assess internal consistency reliability (29). However, recent research shows that single items that are content valid perform comparably and consistently with longer measures for the same constructs (29). The challenge then becomes finding or creating items that appropriately reflect the breadth of the construct being measured. This was particularly difficult for our perceived organizational support item as no single item measure has previously been used and validated. Our item may only cover the perceived work valuation aspect of POS and not the personal value and importance to the organization. Further research is needed to determine if the value to the organization changes based on the WOW-cart intervention and not just the value of their work.

Third, because our intervention was multi-faceted, containing both tangible nutritional items and socialization, we are unable to parse out the independent contributions to energy and POS from each. Future research would benefit from engaging in independent measurement of the quality of the socialization and the utilization of nutritional items. Again, as with our first limitation, we strongly advise careful consideration of the burden of time and effort put upon HCWs to measure their work experience intra-workday and in their point-of-care areas. Further, our *post-hoc* RSA findings suggest that future research would benefit from considering a curvilinear effect for resource gains on engagement and well-being.

Fourth, our cross-sectional single time point data creates some limitation. We are unable to make claims about how long these improvements in energy and POS. Future research would benefit from designing a longitudinal follow-up at multiple time points to determine the durability of these improvements and any additional resource benefits beyond the initial gains HCWs experienced from the intervention. We also are unable to determine if the WOW cart intervention consistently has the same effect or if it has a novelty that wears off. We conducted a *post-hoc* independent samples *t*-test using instance (i.e., 1st or 2nd time) experiencing the WOW cart as the grouping variable to examine the differences in changes and levels of energy, POS, and work engagement for the subgroup of 30 HCWs who encountered the WOW cart twice and reported their experiences. Pre- and post-WOW cart levels of energy, POS, and work engagement were not significantly different. Neither was the change scores from pre- to post-WOW cart levels of energy, POS, and work engagement. These results are not exhaustive but suggest that the WOW cart is not just a novelty. Future research would benefit from a longer multi-instance examination of the effects of short minimally invasive interventions such as the WOW cart.

Fifth, our study does not have a comparison group. We are not able to show how HCWs experiencing the WOW cart compare to a group of HCWs who do not experience the WOW cart. Consequently, it is difficult to determine where the changes observed in HCWs states of well-being and personal resources are the result of the intervention or other factors. This largely is a result of limitation in measurement capability when striving to be minimally invasive. Future research would benefit from including a control group when measuring the effectiveness of well-being interventions. Areas of an institution could be grouped by similarity of work tasks and recruit participants from both areas where one receives the WOW cart and another does not.

Our limitations identify that our findings should be interpreted with caution, recognizing potential bias and methodological challenges. As such, our study can best be described as providing initial and promising evidence for the benefit of a micro-break intervention to improve personal resources and well-being. Future research would benefit from engaging in a more robust independent measurement of pre- and post-intervention. However, we remind researchers that extreme care must be taken to not put an unreasonable load upon HCWs who are volunteering to participate in research studies while working when patient care and other work demands are plentiful.

### 4.4 Conclusion

We tested the effectiveness of delivering external resources of small nutritional items and positive socialization as a micro-break intervention for HCWs. We found that the WOW Cart was associated with improvements in ratings of HCW energy, POS, and work engagement. In a population whose well-being is critical for the health of our nation, our HCWs, a micro-break intervention may provide more than just a free snack: it ultimately might be a key contributor to building and supporting a culture of well-being in the workplace (1–6).

## Data Availability

The datasets presented in this study can be found in online repositories. The names of the repository/repositories and accession number(s) can be found below: the data associated with this study is available in the OSF repository at https://osf.io/e6w5f/?view_only=61a31552ce494ec68954b1575508b57b.
